# Data assimilation for estimating time-varying reproduction numbers

**DOI:** 10.1098/rsif.2025.0131

**Published:** 2025-12-17

**Authors:** Han Yong Wunrow, Sen Pei, Jeffrey Shaman, Marc Spiegelman

**Affiliations:** 1 Department of Applied Physics and Applied Mathematics, Columbia University, New York, NY, USA; 2 Department of Environmental Health Sciences, Columbia University, New York, NY, USA; 3 Department of Earth and Environmental Sciences, Columbia University, New York, NY, USA; 4 Columbia Climate School, New York, NY, USA

**Keywords:** reproduction number, data assimilation, time-varying parameters, infectious disease dynamics, Life Sciences–Mathematics interface, biomathematics, computational biology

## Abstract

The time-varying basic reproduction number, $R_{0}(t)$, is a key epidemiological metric that quantifies the transmissibility of an infectious pathogen at time t. Accurate estimation and uncertainty quantification of $R_{0}(t)$ are crucial for understanding disease dynamics and informing public health decision-making. In this study, we evaluate six methods for estimating $R_{0}(t)$ using synthetic data generated from a stochastic Susceptible-Infected-Recovered (SIR) model with imposed changes to pathogen transmissibility and empirical COVID-19 case data. The methods include ensemble filter methods and inflation techniques, which are employed to mitigate covariance underestimation and filter divergence. For synthetic data, we compare the ensemble adjustment Kalman filter (EAKF) with no inflation, fixed inflation, and adaptive inflation, and the ensemble square root smoother (EnSRS) with adaptive inflation. For empirical data, we also compare with EpiEstim and EpiFilter. Our results demonstrate that the EAKF and EnSRS methods with adaptive inflation outperform other approaches in accurately estimating $R_{0}(t)$, particularly in scenarios with abrupt changes in transmission rates. The adaptive inflation techniques effectively address covariance underestimation and filter divergence, leading to more robust and reliable estimates of $R_{0}(t)$. These findings highlight the potential of adaptive inflation methods for improving the accuracy of time-varying parameter inference, contributing to more effective public health responses.

## 1. Introduction

The time-varying basic reproduction number, denoted $R_{0}(t)$, is the expected number of secondary cases caused by an infectious individual in a well-mixed population of susceptible individuals at time t. Due to high heterogeneity in infectious disease transmission and behavioural dynamics, $R_{0}(t)$ varies spatiotemporally [1,2]. As such, the estimation of $R_{0}(t)$ is crucial for understanding the trajectory of an outbreak and for implementing effective control measures. In situations where the transmissibility of the pathogen undergoes changes due to the emergence of new variants, human behaviour changes, or non-pharmaceutical interventions, $R_{0}(t)$ provides valuable insights into the effectiveness and feasibility of these interventions [2]. Monitoring $R_{0}(t)$ enables public health officials to assess the impact of control measures in real time and to adjust strategies accordingly. Additionally, understanding the temporal variations in $R_{0}(t)$ can help anticipate the potential resurgence of disease and inform proactive measures to prevent further spread.

Another related but different quantity is the effective reproduction number, denoted $R_{e}(t)$, defined as the expected number of secondary cases caused by an infectious individual in a well-mixed population of both susceptible and non-susceptible individuals at time t. Both $R_{0}(t)$ and $R_{e}(t)$ play distinct roles in epidemiological analysis and public health decision-making. Since $R_{0}(t)$ represents the transmission potential of the pathogen in a fully susceptible population, it can provide insights into the overall magnitude of an outbreak. It serves as a fundamental parameter for epidemic modelling and forecasting, guiding preparedness efforts and resource allocation. On the other hand, $R_{e}(t)$ reflects the transmissibility of the disease in the current population context, incorporating the accumulation of immunity. Therefore, while $R_{0}(t)$ informs long-term planning and initial response strategies, $R_{e}(t)$ offers a real-time assessment of transmission intensity, guiding immediate control measures and outbreak management.

There are several methods for estimating $R_{0}(t)$ and $R_{e}(t)$. Two common approaches are through the use of either the renewal equation [3,4] or compartmental models [5–7]. The renewal equation provides a theoretical framework for estimating $R_{e}(t)$ by considering the generation interval distribution, which is the time distribution of infectious contacts between infector–infectee pairs. Since these infectious contact events are rarely observed, the serial interval is often used in its place. The serial interval is the time of symptom onset between infector–infectee pairs. As noted by Gostic *et al*. [8], the serial interval distribution may not be a suitable substitute for the generation time distribution, particularly when there is a long latent period.

Alternatively, compartmental models, such as the Susceptible-Infected-Recovered (SIR) model, divide a population into disease states and model the flow between compartments usually with ordinary or stochastic differential equations. Both $R_{e}(t)$ and $R_{0}(t)$ can be computed using the flow rate parameters and model state values, commonly with the next-generation matrix [9]. Although both are viable approaches to compute reproduction numbers, compartmental models have the advantage of distinguishing disease states. There is also an implicit connection between a compartmental model and its corresponding generation interval distribution [10].

A popular approach to calibrating compartmental models is with ensemble filters. Ensemble filters use an empirical distribution of model state vectors to estimate the relation between observations and model state variables and parameters in a sequential manner. Current ensemble filter methods often face challenges with underestimating uncertainty and capturing temporal trends when there are abrupt changes in parameters. This is applicable to modelling epidemics when there is a shift in transmission dynamics caused by interventions or the emergence of new variants. In the worst case, ensemble filters can experience filter divergence where the empirical distribution becomes degenerate. Several heuristics have been developed to address these challenges, namely multiplicative covariance inflation [11], which simply increases the prior ensemble variance by a factor. The inflation factor is usually determined via empirical tuning, which can be an expensive procedure. Anderson [12] proposed a data-driven adaptive inflation scheme that computes an inflation factor using a Bayesian approach that varies spatially and temporally. Another approach to obtain more robust posteriors is to apply a fixed-lag smoother where the model state is estimated using observations a fixed number of time steps into the future. There are many ensemble filter and fixed-lag smoothing techniques. For a recent review of ensemble filter methods, the reader is referred to [13] and references therein. Here we perform inference using the ensemble adjustment Kalman filter (EAKF) [14] and ensemble square root smoother (EnSRS) [15].

In addition to our work, other advanced methods for estimating time-varying reproduction numbers have been developed. EpiFilter [16] is a recursive Bayesian smoother based on Kalman filtering theory in which the reproduction number is modelled as a hidden Markov state on a fixed discrete grid. Steyn & Parag [17] later expanded this approach by developing a Bayesian framework to automatically select smoothing parameters for both EpiFilter and EpiEstim, which can otherwise negatively impact $R_{e}(t)$ inference by producing overconfident estimates. Storvik *et al*. [18] developed a sequential Monte Carlo (SMC) approach on top of a stochastic Susceptible-Exposed-Infected-Recovered (SEIR) model to estimate a daily-varying reproduction number using multiple data streams from the COVID-19 pandemic in Norway. Similarly, Koyama *et al*. [19] also devised a SMC method with a state-space method based on a discrete-time variant of the Hawkes process to detect changes in the reproduction number. Yang *et al*. [20] proposed a Bayesian data assimilation framework (DARt) that uses a particle filter to jointly estimate infections and $R_{e}(t)$, which enables estimation of abrupt changes and uncertainty. For other contributions using Bayesian techniques with different modelling approaches, see e.g. [21–23].

These methods predominantly use SMC methods (also known as particle filters) that represent the probability distribution of the system’s state with a set of weighted particles. While particle filters are robust and can handle non-Gaussian and nonlinear systems, they can be computationally intensive. By contrast, the EAKF used in our study uses a deterministic and computationally efficient update step that avoids the resampling noise and potential spurious correlation issues of particle filters, making it a highly practical choice for real-time epidemiological tracking, especially when needing to respond to abrupt changes in transmission. A comparative analysis against these particle-based approaches, particularly those incorporating adaptive mechanisms, would be valuable for a complete understanding of the strengths and weaknesses of different data assimilation strategies in this context.

In this study, we evaluate six methods for estimating the time-varying basic reproduction number, $R_{0}(t)$, using 47 871 parameter scenarios of synthetic data generated from a stochastic SIR model all with abrupt increases in $R_{0}(t)$. Specifically, we compare the EAKF with no inflation, a fixed inflation factor, and Anderson’s adaptive inflation scheme, the EnSRS with adaptive inflation, EpiEstim [3] and EpiFilter [16]. In this paper, we contribute a systematic comparison and demonstration of adaptive inflation’s utility in significantly enhancing the accuracy and uncertainty quantification of $R_{0}(t)$ estimates.

## 2. Material and methods

### 2.1. Synthetic data

We generated synthetic data using a stochastic SIR model simulated by the following stochastic difference equations and initial conditions:

(2.1) $$\left\{\begin{array}{ll} dSI & \sim \text{Pois}\left(\frac{\beta (t)S(t)I(t)}{N}\tau \right)\\ dIR & \sim \text{Pois}\left(\frac{I(t)}{D}\tau \right)\\ S(t+\tau ) & =S(t)-dSI\\ I(t+\tau ) & =I(t)+dSI-dIR\\ R(t+\tau ) & =R(t)+dIR\\ i(t+\tau ) & =dSI\\ S(0) & =S_{0}\\ I(0) & =I_{0} \end{array}\right.$$

where S(t),I(t) and R(t) are the number of susceptible, infectious and removed individuals at time t, respectively, N=S+I+R is the total population size, β(t) is the transmission rate at time t and D is the mean infectious period, which we assume to be constant. i(t) is the incidence at time t and is the observed state of this system. The model was simulated using the Poisson τ-leaping algorithm with a time step size of τ=1. This algorithm is analogous to the forward Euler method for deterministic systems and similar to an Euler–Maruyama type discretization [24].

The time-varying basic reproduction number for this compartmental model is given by

(2.2) $$R_{0}(t)=\beta (t)D.$$

#### 2.1.1. Change point

We imposed a sigmoid form for β(t) given by

(2.3) $$\beta (t)=\beta_{0}+\frac{\beta_{1}-\beta_{0}}{1+e^{-k\left(t-t_{mid}\right)}},$$

where $\beta_{0}$ is the starting transmission rate, $\beta_{1}$ is the ending transmission rate, $t_{\text{mid}}$ is the time at which the transmission rate is $\left(\beta_{0}+\beta_{1}\right)/2$ (i.e. inflection point of the sigmoid function), k controls the steepness of the sigmoid function, and $\beta_{0},\beta_{1},t_{\text{mid}},k>0$.

#### 2.1.2. Observation error variance

To simulate the inherent stochastic uncertainties associated with empirical observations, we add a Gaussian noise term to the daily case counts generated from equation (2.1),

(2.4) $$i_{j}^{\text{obs}}=i_{j}^{\text{true}}+\eta_{j},j=1,\ldots ,T,\;\eta_{j}\sim 𝒩\left(0,\gamma_{j}\right),$$

where

(2.5) $$\gamma_{j}=\text{max}\left\{\gamma_{min},\frac{{\left(i_{j}^{\text{true}}\right)}^{2}}{\gamma_{\text{scale}}}\right\}$$

with $\gamma_{\text{min}}=10,\gamma_{\text{scale}}=50$, and the subscript j denoting the observation at time $t_{j}=\tau j$. Within the context of data assimilation, $\gamma_{j}$ is referred to as the observation error variance (OEV). This form of OEV scales with ${\left(i_{j}^{\text{true}}\right)}^{2}$ indicating that the uncertainty in the observed case counts increases as the true number of infections increases. This is a reasonable assumption, as larger outbreaks tend to have more reporting errors. The division by $\gamma_{\text{scale}}$ helps ensure that the OEV does not become unreasonably large for very high case counts. The minimum of $\gamma_{\text{min}}$ prevents the OEV from becoming too small when there are very few cases. A small OEV would imply high confidence in the observed case counts, which may not be realistic in the early stages of an outbreak. Similar forms of OEV have successfully been applied to models of infectious disease dynamics [25–29]. The values of $\gamma_{\text{min}}$ and $\gamma_{\text{scale}}$ were chosen empirically, guided by the explored parameter space of the sigmoid parameters and the initial values of the SIR model, to ensure the introduction of a suitable level of noise.

#### 2.1.3. Parameter space

To generate synthetic outbreaks, we performed a grid search to identify sigmoid parameters $\left(\beta_{0},\beta_{1},k,m\right)$ that produced two epidemic curves within the following ranges: $0.275\leq \beta_{0},\beta_{1}\leq 1.275,50\leq m\leq 250,0.1\leq k\leq 0.9$ and D=4 was held constant, resulting in a total of 47 871 synthetic parameter scenarios. All scenarios used a total population size of N=100000 and initial conditions $S_{0}=99\;900$ and $I_{0}=100$.

### 2.2. Ensemble adjustment Kalman filter

Inference was performed using the EAKF [14]. The EAKF is a sequential data assimilation scheme that combines an ensemble of unobserved model states and parameters (representing the distribution of a system’s state variables) with observed data using a deterministic algorithm to iteratively estimate and correct the system state while accounting for uncertainties in both the model and observations. We selected EAKF for this study due to its deterministic update step, which avoids the additional sampling noise inherent in stochastic versions of the ensemble Kalman filter (EnKF), and its demonstrated effectiveness in similar models of infectious disease dynamics [25–29]. Anderson introduces the EAKF in the general multivariate case. Since our observations are one-dimensional, we bypass the matrix algebra of the EAKF and discuss its details in the scalar case. The reader is referred to electronic supplementary material, text S1, along with [14] for details of the multivariate case. The notation used can be found in table 1.

Consider the stochastic dynamics model given by

(2.6) $$v_{j+1}=\Psi \left(v_{j}\right),j=0,\ldots ,T-1,\;v_{0}\sim 𝒰(a,b)$$

where $v_{j}$ is the random vector representing the model states and parameters at time $t_{j}$,

(2.7) $$v_{j}={\left(S\left(t_{j}\right),I\left(t_{j}\right),R\left(t_{j}\right),\beta \left(t_{j}\right),D,i\left(t_{j}\right)\right)}^{⊤}.$$

Ψ(⋅) is the stochastic SIR model given by equation (2.1) so

(2.8) $$\Psi \left({\left(S\left(t_{j}\right),I\left(t_{j}\right),R\left(t_{j}\right),\beta \left(t_{j}\right),D,i\left(t_{j}\right)\right)}^{⊤}\right)={\left(S\left(t_{j+1}\right),I\left(t_{j+1}\right),R\left(t_{j+1}\right),\beta \left(t_{j}\right),D,i\left(t_{j+1}\right)\right)}^{⊤}.$$

We also employ a uniform prior for $v_{0}$ instead of a Gaussian prior to reflect a lack of strong prior knowledge about the initial conditions, avoiding a potential bias towards the mean. Now consider the data model given by

(2.9) $$y_{j+1}=Hv_{j+1}+\eta_{j+1},j=0,\ldots ,T-1,\;\eta_{j+1}\sim 𝒩\left(0,\gamma_{j+1}\right),$$

where $y_{j+1}$ is an observation random variable representing the truth at time $t_{j+1},H=(0,0,0,0,0,1)$ is our linear observation operator that maps the latent model state into the observation space, and $\eta_{j+1}$ is given by equation (2.5).

The EAKF approximates the posterior distributions $P\left(v_{j+1}|\left\{y_{1},\ldots ,y_{j},y_{j+1}\right\}\right)$ using an ensemble of M members, denoted by $v_{j}^{\left(m\right)}$ where m=1,…,M. Each ensemble member is equally weighted and represents a possible state of the system at time $t_{j}$. Following the notation from Sanz-Alonso *et al*. [30], the EAKF algorithm can be decomposed into two steps:

(2.10) $$\begin{array}{c} \text{Prior}/\text{Forecast}\left\{\begin{array}{ll} {\hat{v}}_{j+1}^{\left(m\right)} & =\Psi \left(v_{j}^{\left(m\right)}\right),\;m=1,\ldots ,M\\ {\hat{y}}_{j+1}^{\left(m\right)} & =H{\hat{v}}_{j+1}^{\left(m\right)}\\ {\hat{m}}_{j+1} & =\frac{1}{M}\sum_{m=1}^{M}{\hat{v}}_{j+1}^{\left(m\right)},\\ {\hat{C}}_{j+1} & =\frac{1}{M-1}\sum_{m=1}^{M}\left({\hat{v}}_{j+1}^{\left(m\right)}-{\hat{m}}_{j+1}\right){\left({\hat{v}}_{j+1}^{\left(m\right)}-{\hat{m}}_{j+1}\right)}^{⊤},\\ {\hat{\sigma }}_{j+1}^{2} & =H{\hat{C}}_{j+1}H^{⊤} \end{array}\right. \end{array}$$

(2.11) $$\text{Posterior}/\text{Analysis}\left\{\begin{array}{ll} \alpha & ={\left[\gamma_{j+1}{\left(\gamma_{j+1}+{\hat{\sigma }}_{j+1}^{2}\right)}^{-1}\right]}^{1/2},\\ Hm_{j+1} & =\alpha^{2}H{\hat{m}}_{j+1}+\left(1-\alpha^{2}\right)y_{j+1}^{†},\\ y_{j+1}^{\left(m\right)} & =\alpha \left({\hat{y}}_{j+1}^{\left(m\right)}-H{\hat{m}}_{j+1}\right)+Hm_{j+1},\\ v_{j+1}^{\left(m\right)} & ={\hat{v}}_{j+1}^{\left(m\right)}+{\hat{C}}_{j+1}H^{⊤}{\left({\hat{\sigma }}_{j+1}^{2}\right)}^{-1}\left(y_{j+1}^{\left(m\right)}-{\hat{y}}_{j+1}^{\left(m\right)}\right). \end{array}\right.$$

The hat notation is used to indicate ensemble members and statistics for the prior distribution $P\left({\text{v}}_{\text{j}+1}|\left\{y_{1},\ldots ,y_{j}\right\}\right)$. Variables without the hat notation are for the posterior distribution $P\left({\text{v}}_{\text{j}+1}|\left\{y_{1},\ldots ,y_{j},y_{j+1}\right\}\right).y_{j+1}^{†}$ is the actual observed value as opposed to the random variable $y_{j+1}$.

Note that the equations in (2.11) use the two-step framework of the analysis step in Anderson [31] where we first update the ensemble posterior of the observed variable and then update our latent state variables and parameters. As Anderson points out, the adjustment to compute the posterior ensemble ${\left\{v_{j+1}^{\left(m\right)}\right\}}_{m=1}^{M}$ is equivalent to regressing ${\left\{{\hat{v}}_{j+1}^{\left(m\right)}\right\}}_{m=1}^{M}$ against ${\left\{{\hat{y}}_{j+1}^{\left(m\right)}\right\}}_{m=1}^{M}$, as ${\hat{C}}_{j+1}H^{⊤}{\left({\hat{\sigma }}_{j+1}^{2}\right)}^{-1}$ is the covariance between ${\left\{{\hat{y}}_{j+1}^{\left(m\right)}\right\}}_{m=1}^{M}$ and ${\left\{{\hat{v}}_{j+1}^{\left(m\right)}\right\}}_{m=1}^{M}$ divided by the variance of ${\left\{{\hat{y}}_{j+1}^{\left(m\right)}\right\}}_{m=1}^{M}$, which is the ordinary least squares estimator.

The last equation in (2.11) can also be rewritten as

(2.12) $$v_{j+1}^{\left(m\right)}=A\left({\hat{v}}_{j+1}^{\left(m\right)}-{\hat{m}}_{j+1}\right)+m_{j+1}$$

where

(2.13) $$A=I+{\left({\overset{ˆ}{\sigma }}_{j+1}^{2}\right)}^{-1}\left(\alpha -1\right){\tilde{C}}_{j+1}.$$

${\tilde{C}}_{j+1}$ is a matrix with all-zero entries except for the last column, which is identical to the last column of ${\hat{C}}_{j+1}$. Here A satisfies

(2.14) $$C_{j+1}=A{\hat{C}}_{j+1}A^{\text{T}}={\left[{\hat{C}}_{j+1}^{-1}+H^{\text{T}}\gamma_{j+1}^{-1}H\right]}^{-1}.$$

### 2.3. Fixed inflation

To mitigate the issue of covariance underestimation and prevent filter divergence, a common approach is to employ multiplicative covariance inflation [11]. This method involves scaling the deviation of each ensemble member $v_{j+1}^{\left(n\right)}$ from the ensemble mean $m_{j+1}$ by a fixed inflation factor λ, effectively increasing the ensemble spread,

(2.15) $$v_{j+1}^{\left(m\right)}\leftarrow \text{diag}\left(\sqrt{\lambda }\right)\left(v_{j+1}^{\left(m\right)}-m_{j+1}\right)+m_{j+1}.$$

The vector λ typically has different fixed inflation factors for each state variable or parameter and is typically determined using empirical tuning.

### 2.4. Adaptive inflation

The adaptive inflation algorithm proposed by Anderson [12] treats the inflation factor, denoted as $\lambda_{j+1}$, as a random variable that varies temporally. The core idea behind this method is to dynamically adjust the ensemble variance to better match the observed data. If the model forecasts are too confident and consistently miss the observations, it suggests that model uncertainty is underestimated. Adaptive inflation corrects this by increasing the ensemble variance based on the ‘innovation’, or the difference between the observation and the model’s prior mean. This data-driven adjustment helps to prevent filter divergence and improves the reliability of the state estimates. Here we describe the adaptive inflation algorithm.

(2.16) $$v_{j+1}^{\left(m\right)}\leftarrow \text{diag}\left(\sqrt{\lambda_{j+1}}\right)\left(v_{j+1}^{\left(m\right)}-m_{j+1}\right)+m_{j+1}.$$

Again, the vector $\lambda_{j+1}$ typically has different adaptive inflation factors for each state variable or parameter. Similar to the two-step framework of the analysis step in the EAKF, the inflation factor for the observation variable, denoted $\lambda_{j+1}$ is computed first, and then all other inflation factors are computed.

The inflation factor is computed based on the difference between the observation and the prior mean of the observed variable, commonly referred to as the innovation, $d_{j+1}$,

(2.17) $$d_{j+1}=y_{j+1}-H{\hat{m}}_{j+1}.$$

The expected value of the squared innovation, $E\left[d_{j+1}^{2}\right]$, is related to the observation error variance, $\gamma_{j+1}^{2}$, and the true prior variance of the observation variable, ${\hat{\sigma }}_{j+1}^{2(\text{true})}$, as

(2.18) $$E\left[d_{j+1}^{2}\right]=\gamma_{j+1}^{2}+{\hat{\sigma }}_{j+1}^{2(\text{true})}.$$

Anderson [12] assumes a linear relationship between the true prior variance and the sample variance from the ensemble, ${\hat{\sigma }}_{j+1}^{2(\text{true})}=\lambda_{j+1}{\hat{\sigma }}_{j+1}^{2}$, the inflation factor can be estimated as

(2.19) $$\lambda_{j+1}=\frac{E\left[d_{j+1}^{2}\right]-\gamma_{j+1}^{2}}{{\hat{\sigma }}_{j+1}^{2}}.$$

Anderson’s adaptive inflation algorithm uses a Bayesian approach to update the inflation factor [12]. The prior distribution for the inflation factor is assumed to be Gaussian, with mean $\mu_{\lambda }$ and variance $\sigma_{\lambda }^{2}$, and the likelihood function is computed from the innovation statistics. We initialize the prior with $\mu_{j}=1.01$ and $\sigma_{\lambda }^{2}=0.0001$. The posterior distribution is then obtained by combining the prior and likelihood using Bayes’ theorem so that

(2.20) $$p\left(\lambda_{j+1}|d_{j+1}^{†}\right)\propto \frac{1}{2\pi \sigma_{\lambda }\theta }\text{exp}\left[-\frac{{\left(\lambda_{j+1}-\mu_{\lambda }\right)}^{2}}{2\sigma_{\lambda }^{2}}-\frac{{\left(d_{j+1}^{†}\right)}^{2}}{2\theta^{2}}\right],$$

where $\theta =\gamma_{j+1}^{2}+\lambda_{j+1}{\hat{\sigma }}_{j+1}^{2}$ and $d_{j+1}^{†}=y_{j+1}^{†}-H{\hat{m}}_{j+1}$. Similar to the iterative nature of the EAKF, the posterior at time $t_{j+1}$ becomes the prior at time $t_{j+2}$.

The updated inflation value is taken as the mode of the posterior distribution using a Laplace approximation of the posterior. This is done by first approximating the likelihood with a Taylor expansion and keeping only the linear terms. The derivative of the resulting approximate posterior is then set to zero, leading to a quadratic equation in $\lambda_{j+1}$. Two solutions are obtained, and the one that is closest to the previous inflation factor $\lambda_{j}$ is selected. To compute the inflation factors for the latent state variables and parameters, we assume an equivalent correlation structure between the state variables and the inflation, such that

(2.21) $$\lambda_{j+1}(k)={\left(\frac{\lambda_{j+1}^{1/2}-1}{r}+1\right)}^{2},$$

where $r=\text{corr}\left(y_{j+1},v_{j+1}(k)\right)$. A comprehensive description of the adaptive inflation algorithm and its implementation is provided in Anderson [12].

Anderson’s adaptive inflation algorithm provides a framework for estimating and updating the inflation factor in the EAKF. By considering the inflation factor as a random variable and using a Bayesian approach, the algorithm allows for the adaptive adjustment of the inflation factor based on the observed data, leading to improved estimation of the true variance. For a geometrical interpretation and further details of the algorithm, the reader is referred to [12,32].

### 2.5. Ensemble square root smoother

The fixed-lag EnSRS proposed by Whitaker & Compo [15] approximates the posterior distribution of the model state at time $t_{j}$ given observations up until time $t_{j+l},P\left(v_{j}|\left\{y_{1},\ldots ,y_{j+l}\right\}\right)$, for some fixed time lag l. For this analysis, we use l=10. The approximation of this posterior distribution is done in an iterative manner starting with the lag l=0.

The smoothing Kalman gain uses a cross-covariance matrix between the forecast of the Kalman filter update equation for time $t_{j+l}$ and the lag l−1 Kalman smoother analysis for time $t_{j}$. This is derived in a least squares sense and is used to produce the smoothed analysis ensemble. The cross-covariance matrix and Kalman gain are also used to update the analysis covariance matrix.

(2.22) $$v_{j|j+l}=v_{j|j+l-1}-{\tilde{K}}_{j|j+l}{\overset{ˆ}{y}}_{j},$$

where the subscript notation j∣j+l refers to conditional random variables at time $t_{j}$, which is conditioned on observations up until time $t_{j+l}$.


(2.23) $${\tilde{K}}_{j|j+l}={\left(1+\sqrt{\frac{\gamma_{j+l}}{H{\hat{C}}_{j+l|j+l-1}H^{⊤}+\gamma_{j+l}}}\right)}^{-1}K_{j|j+l}.$$

### 2.6. EpiEstim

The method from Cori *et al*. [3] estimates the effective reproduction number $R_{e}(t)$ as

(2.24) $$R_{e}(t)=\frac{i_{t}}{\sum_{s=1}^{t}i_{t-s}w_{s}},$$

where $i_{t}$ is the number of infection incidents at time t and $w_{s}$ is the generation interval distribution probability that s days separate the infection incident and a secondary case. Since these infectious contact events are rarely observed, the serial interval is often used in its place. In EpiEstim, it is assumed that the transmission process follows a Poisson process such that the probability someone infected at time t−s infects someone new at time t is $R_{e}(t)w_{s}$. EpiEstim also incorporates a sliding window of size Δ to smooth estimates. It is assumed that the effective reproduction number $R_{e}(t)=R_{t,\Delta }$ is constant for t−Δ+1,…,t.

Prior:

(2.25) $$R_{t,\Delta }\sim \text{Gamma}(a,b).$$

Likelihood:

(2.26) $$i_{t}|i_{0:t-1},w,R_{t,\Delta }\overset{\text{ind}}{\sim }\text{Pois}\left(R_{t,\Delta }\Lambda_{t}\right),$$

where $\Lambda_{t}=\sum_{s=1}^{t}i_{t-s}w_{s}$ so

(2.27) $$P\left(i_{t-\Delta +1:t}|i_{0:t-\Delta },w,R_{t,\Delta }\right)=\prod_{s=t-\Delta +1}^{t}P\left(i_{s}|i_{0:s-1},w,R_{t,\Delta }\right).$$

It follows that the posterior distribution is

(2.28) $$R_{t,\Delta }|i_{0:t-\Delta },w\sim \text{Gamma}\left(a+\sum_{s=t-\Delta +1}^{t}i_{s},b+\sum_{s=t-\Delta +1}^{t}\Lambda_{s}\right),$$

Note that this derivation did not yet take into account the uncertainty of the generation (serial) interval distribution $w_{s}$. However, the user specifies the mean and standard deviation of the generation (serial) interval distribution so that $w_{s}\sim \text{Gamma}(\alpha ,\beta )$.

Connecting this to a SIR compartmental model, the intrinsic generation-time interval of an SIR model is

$$w_{s}\sim \text{Exponential}(1/\text{D})=\text{Gamma}(1,1/\text{D})$$

as noted by Wallinga & Lipsitch [10]. We also have

(2.29) $$R_{e}\left(t\right)=\beta \left(t\right)D\frac{S\left(t\right)}{N}.$$

It follows that

(2.30) $$R_{0}\left(t\right)=R_{e}\left(t\right)\frac{N}{S\left(t\right)}.$$

The supplementary material of [3] outlines a choice of the time window Δ; however, it suggests a Δ that minimizes the number of cases within the time window.

### 2.7. EpiFilter

The EpiFilter method from Parag [16] estimates $R_{e}(t)$ using the renewal equation (2.26), similar to EpiEstim. However, EpiFilter performs inference using a recursive Bayesian smoother derived from Kalman filtering theory, similar to our approach. This deterministic approach uses a forward–backward algorithm to compute the filtering posterior distribution, $p\left(R_{e}(t)|i_{0:t}\right)$, and the smoothing posterior distribution, $p\left(R_{e}(t)|i_{0:T}\right)$. In this work, we compare EpiFilter’s filtering posterior distribution against our EAKF methods.

EpiFilter models $R_{e}(t)$ as a discrete-time Markov process that evolves over a fixed discrete grid, $ℛ:=\left\{R_{\text{min}},R_{\text{min}}+\delta ,\ldots ,R_{\text{max}}\right\}$ where $\delta :=m^{-1}\left(R_{\text{max}}-R_{\text{min}}\right)$. The state space model is given by

(2.31) $$R_{e}\left(t\right)=R_{e}\left(t-1\right)+\left(\eta \sqrt{R_{e}\left(t-1\right)}\right)\epsilon_{t-1}.$$

Here, $\epsilon_{t-1}\sim 𝒩(0,1)$, and $\left(m,R_{\text{min}},R_{\text{max}},\eta \right)$ are hyperparameters chosen by the user. The parameter η acts as a smoothing hyperparameter, with smaller values leading to smoother estimates. The authors note that η=0.1 serves as a general heuristic that balances accuracy and detection of abrupt temporal shifts in $R_{e}(t)$. For further details of EpiFilter, the reader is referred to the original publication [16].

### 2.8. Performance evaluation

We generated synthetic data for 47 871 parameter scenarios with 100 realizations for each method. To evaluate each method we then calculated the root mean square error (RMSE), the continuous rank prediction score (CRPS), as well as the Kullback–Leibler (KL) divergence $D_{KL}(p\|q)$ and the quadratic Wasserstein distance $W_{2}(p,q)$ between the empirical measures of the posterior predictive p and of the data distributions q. All metrics are averaged over time from $t_{1}$ to $t_{J}$. The RMSE was compared with the posterior ensemble members of the time-varying basic reproduction number $R_{0}^{\left(m\right)}\left(t_{j}\right)$ with the truth $R_{0}^{†}\left(t_{j}\right)$ and is given by

(2.32) $$\text{RMSE}=\frac{1}{J}\sum_{j=1}^{J}{\left(\frac{1}{M}\sum_{m=1}^{M}{\left(R_{0}^{†}\left(t_{j}\right)-R_{0}^{\left(m\right)}\left(t_{j}\right)\right)}^{2}\right)}^{1/2}.$$

To assess the agreement between the posterior predictive distribution p and the data distribution q of daily case counts, we compute the following performance metrics:

(2.33) $$D_{KL}(p\|q)=\frac{1}{J}\sum_{j=1}^{J}\sum_{m=1}^{M}p\left(X_{m}\right)\text{log}\left(\frac{p\left(X_{m}\right)}{q\left(Y_{m}\right)}\right),$$

(2.34) $$W_{2}(p,q)=\frac{1}{J}\sum_{j=1}^{J}{\left(\frac{1}{M}\sum_{m=1}^{M}{\left\|X_{m}-Y_{m}\right\|}^{2}\right)}^{1/2},$$

(2.35) $$\text{CRPS}\left(F,y_{J}^{†}\right)=\frac{1}{J}\sum_{j=1}^{J}\sum_{m=1}^{M-1}{\left(F\left(X_{m}\right)-1_{X_{m}\geq y_{j}^{†}}\right)}^{2}\left(X_{m+1}-X_{m}\right).$$

Here $X_{1},\ldots ,X_{M}$ and $Y_{1},\ldots ,Y_{M}$ are ordered samples from p and q, respectively. F is the empirical cumulative distribution function of the posterior predictive distribution. The posterior predictive distribution is defined as $p\left(i^{\text{sim}}|{\left\{y_{j}^{†}\right\}}_{j=1}^{T-1}\right)=\int p\left(i^{\text{sim}}|\theta \right)p\left(\theta |{\left\{y_{j}^{†}\right\}}_{j=1}^{T-1}\right)$ where $\theta ={\left\{\left(\beta \left(t_{j}\right),D\right)\right\}}_{j=1}^{T-1}$ are the model parameters. The data distribution q was generated by running multiple realizations of the stochastic SIR model with the true model parameters.

The KL divergence and quadratic Wasserstein distance are commonly used to measure the closeness between distributions. Lower values indicate closer distributions. The CRPS is a proper scoring rule for evaluating the accuracy of probabilistic predictions. It measures the difference between the prediction cumulative distribution function (CDF) and the observation $y_{j}^{†}$, summed across all possible values. A lower CRPS indicates a better prediction, with a CRPS of 0 indicating a perfect prediction. Here RMSE measures how well the method recovered the true synthetic $R_{0}(t)$. The KL divergence, quadratic Wasserstein distance, and CRPS measure the dissimilarity between the entire posterior predictive distribution of cases and the true data distribution.

Each of these three metrics was calculated on two different days: the beginning and the end of the second epidemic. The beginning of the second epidemic was defined at the local minimum between the two epidemic curves. The end of the second epidemic was defined as the day after the final epidemic wave has completed where there continues to be zero daily new cases. These dates were chosen to make the comparison between the Kalman filter methods and EpiEstim fair since EpiEstim becomes less robust during periods of low incidence [16]. See the electronic supplementary material for more details on the computation of these two dates.

## 3. Results and discussion

### 3.1. Performance evaluation on synthetic data

The results from our analysis showcase the comparative performance of various methods in estimating $R_{0}(t)$. Among the methods evaluated, the EnSRS with adaptive inflation performs the best with the lowest $R_{0}(t)$ RMSE, $W_{2}(p,q)$ and CRPS at the end of the second epidemic (table 2). Moreover, this method also achieves the lowest $R_{0}(t)$ RMSE and $W_{2}(p,q)$ at the beginning of the second epidemic (table 3). The EAKF with adaptive inflation also demonstrates promising performance, with the lowest $D_{KL}(p\|q)$ and CRPS at the beginning of the second epidemic and comparable metrics to the EnSRS with adaptive inflation at the end of the second epidemic.

The EAKF with adaptive inflation, while exhibiting comparable performance to the no inflation and fixed inflation method at the beginning of the second epidemic, demonstrates its strength as the epidemic progresses. The adaptive inflation method adjusts the ensemble variance and appears to facilitate a more responsive tracking of shifts in $R_{0}(t)$. This observation suggests that adaptive inflation may play a crucial role in maintaining filter accuracy during periods of dynamic epidemiological change, warranting further investigation in future studies. These results also underscore the importance of adaptive inflation techniques in enhancing the accuracy of predictive models for tracking disease transmission dynamics, with implications for informing effective public health interventions.

**Figure 1.**
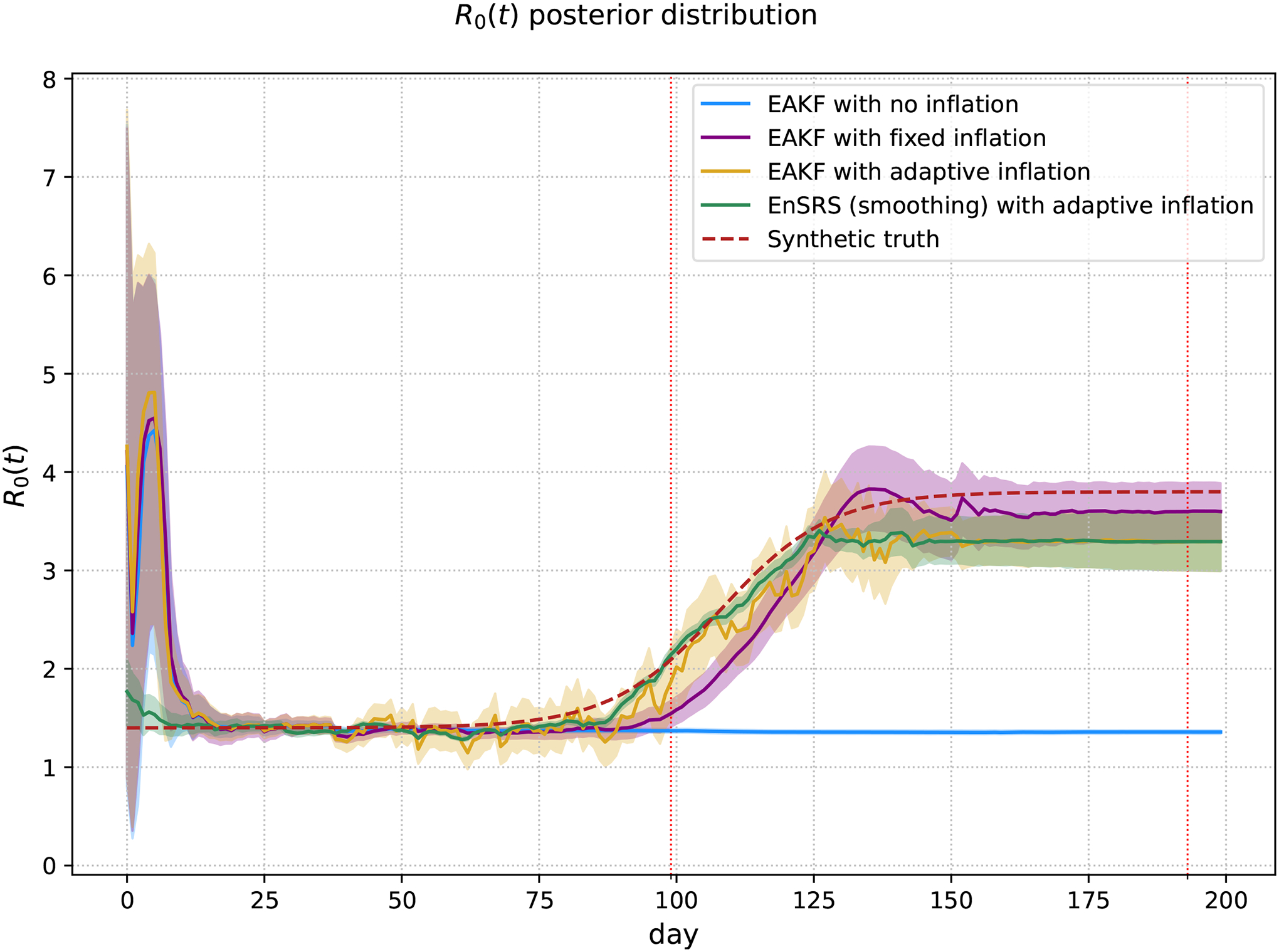
**$R_{0}(t)$** posterior distribution synthetic data. The posterior mean of each method is presented with 95% Crl, represented by the shaded region. The beginning and end of the second epidemic, indicated by the vertical dotted red lines, are day 99 and day 193. The sigmoid parameters for this example were $\beta_{0}=0.35,\beta_{1}=0.95,t_{\text{mid}}=108,k=0.2$.

**Figure 2.**
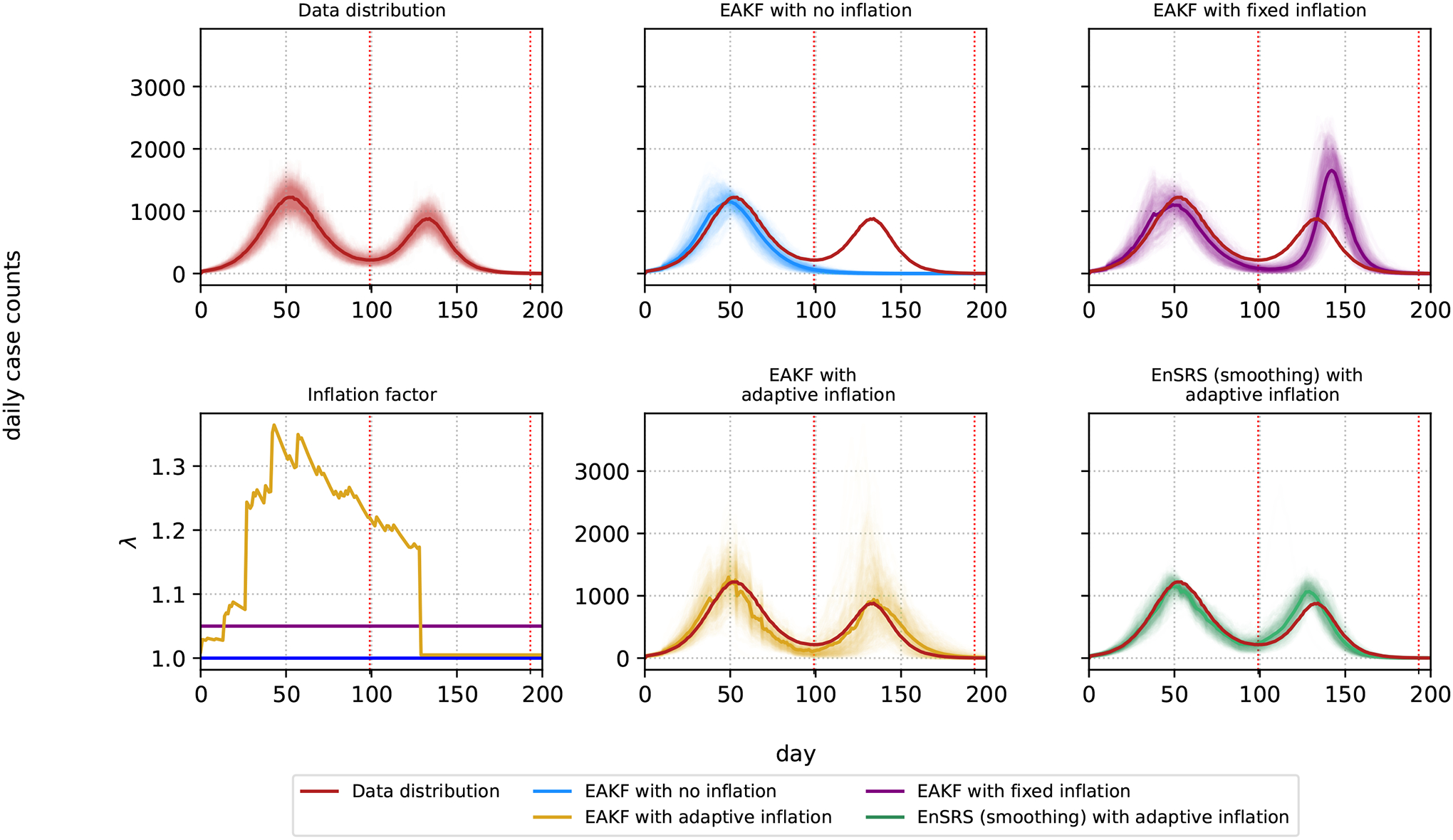
Posterior predictive distribution. Underlying data distribution (in red), inflation λ and posterior predictive distributions for each of the four methods. Each line in the posterior predictive distribution represents one realization. Each solid line represents the mean of the posterior predictive distribution. The beginning and end of the second epidemic, indicated by the vertical dotted red lines, are day 99 and day 193.

Figure 1 shows the posterior mean and 95% credible interval (CrI) of $R_{0}(t)$, as estimated by the four methods. For this particular example, the beginning of the second epidemic was day 99, and the end of the second epidemic was day 193. The sigmoid parameters were $\beta_{0}=0.35,\beta_{1}=0.95,t_{\text{mid}}=108$ and k=0.2. The synthetic truth dashed line in red represents the true $R_{0}(t)$ values used to generate the synthetic data. The EAKF without inflation demonstrates filter divergence, failing to capture the true $R_{0}(t)$ dynamics. The EAKF with fixed and adaptive inflation, as well as the EnSRS with adaptive inflation, show improved performance, with narrower CrIs and closer alignment to the true $R_{0}(t)$, particularly during the second outbreak wave. The adaptive inflation methods appear to be especially adept at capturing the abrupt changes in $R_{0}(t)$, highlighting their potential for robust estimation in scenarios with shifting transmission dynamics. This improved ability to track the true parameter dynamics allows the model to generate posterior predictive distributions (figure 2) that, in turn, more accurately reproduce the timing and amplitude of the second epidemic wave.

Figure 2 provides a visual assessment of the models’ abilities to replicate the observed epidemic dynamics. It displays the posterior predictive distributions for each method, alongside the actual data distribution and the inflation values λ. The posterior predictive distribution is calculated by simulating new streams of daily case counts $i^{\text{sim}}$ from the posterior estimates of the model parameters using the SIR model (equation (2.1)). The close alignment between the posterior predictive distributions of the data distribution with both the EAKF with adaptive inflation and EnSRS with adaptive inflation suggests a superior ability to capture underlying epidemic patterns. By contrast, the EAKF without inflation and with fixed inflation show less satisfactory fits, particularly during the second wave. The inflation plot reveals the dynamic adjustment of λ in response to changes in the epidemic trajectory, highlighting the adaptability of these methods. At the beginning of the second epidemic, the $D_{KL}(p\|q)$ values are: EAKF (no inflation): 1.72, EAKF (fixed inflation): 0.97, EAKF (adaptive inflation): 0.66, EnSRS (adaptive inflation): 1.24. At the beginning of the second epidemic, the $W_{2}(p,q)$ values are: EAKF (no inflation): 0.64, EAKF (fixed inflation): 0.57, EAKF (adaptive inflation): 0.55, EnSRS (adaptive inflation): 0.30. At the end of the second epidemic, the $D_{KL}(p\|q)$ values are: EAKF (no inflation): 10.36, EAKF (fixed inflation): 2.53, EAKF (adaptive inflation): 0.64, EnSRS (adaptive inflation): 1.25. At the end of the second epidemic, the $W_{2}(p,q)$ metric values are: EAKF (no inflation): 1.19, EAKF (fixed inflation): 0.82, EAKF (adaptive inflation): 0.53, EnSRS (adaptive inflation): 0.50.

**Figure 3.**
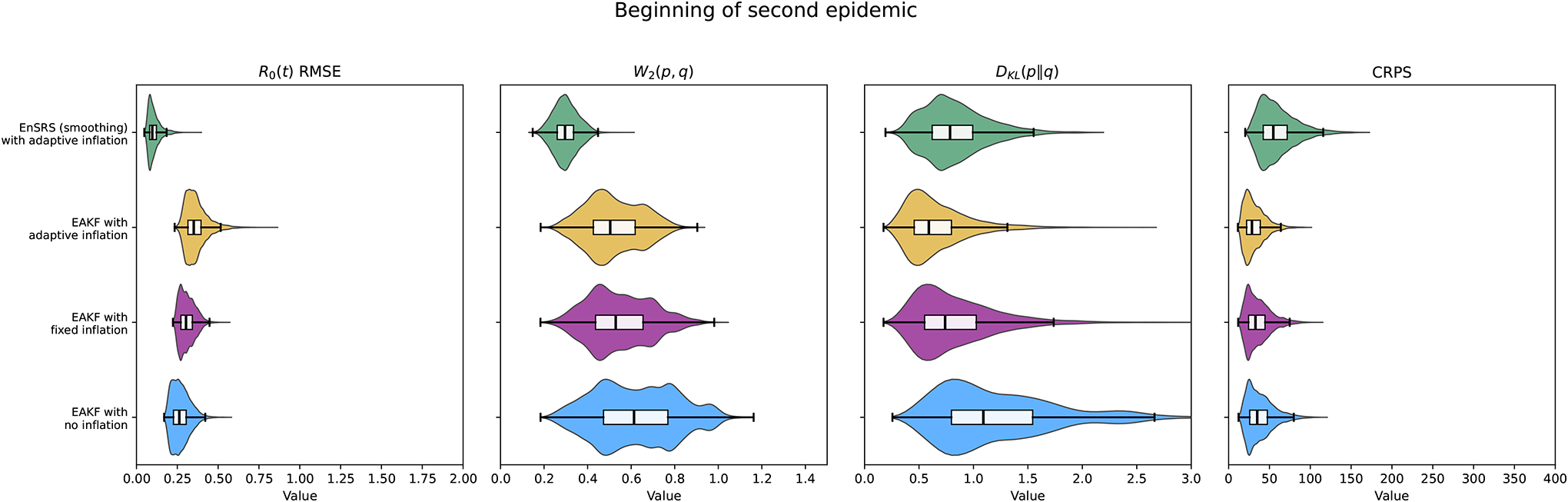

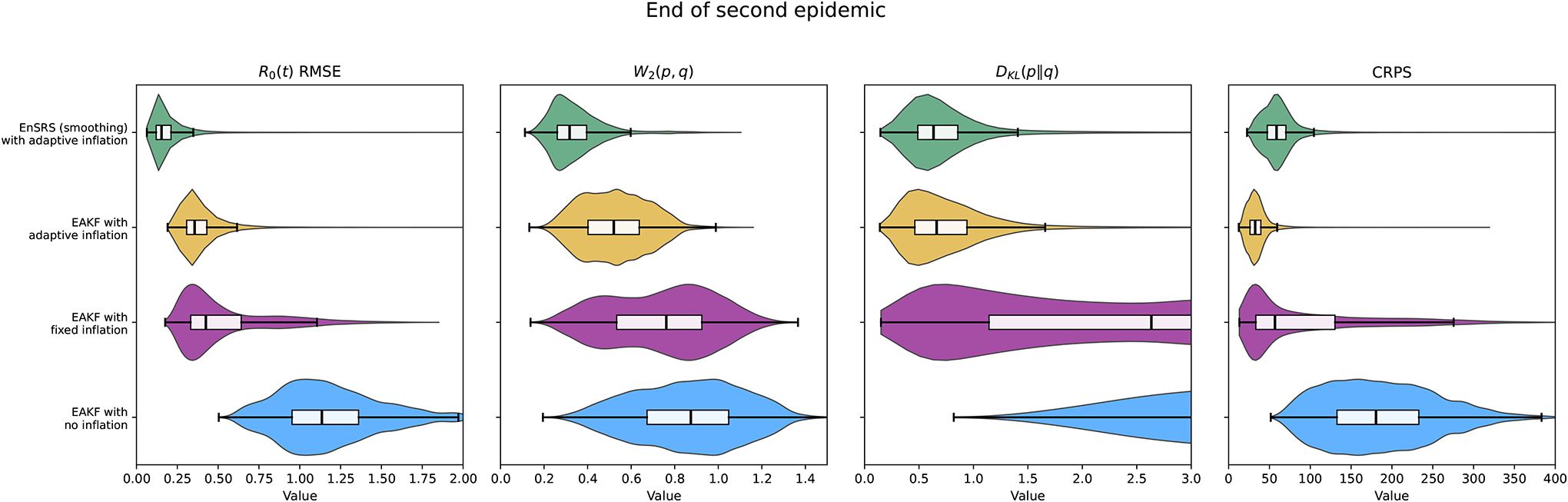
Performance metrics distribution. Violin plots of each performance metric evaluated cumulatively up to the beginning (a) and the end (b) of the second epidemic wave for each of the 47 871 simulated synthetic scenarios and each method. Box plots within violins show the median value and box edges represent the first and third quartiles. The whiskers of the box plot extend to the most distant data points that fall within a range of 1.5 times the interquartile range from the respective quartile.

Figure 3 presents the distributions of each performance metric evaluated cumulatively up to the beginning and up to the end of the second epidemic wave for each of the 47 871 simulated synthetic scenarios. These results are also shown in tables 2 and 3. The histograms reveal that the EAKF and EnSRS methods with adaptive inflation consistently outperform the other methods across almost all metrics at both time points. Notably, the EAKF without inflation exhibits the poorest performance, particularly at the end of the second wave, as indicated by higher RMSE values at the end of the second epidemic.

### 3.2. Performance evaluation on empirical data

To further compare the performance of each method, an empirical evaluation was conducted using real-world COVID-19 daily case data from the Johns Hopkins CSSE COVID-19 Data Repository [33]. In this analysis, the four ensemble Kalman filter and smoother-based methods are compared against two additional established methods: EpiEstim and EpiFilter. Specifically, we used data from Manhattan (New York County), New York from 5 December 2021 to 1 July 2022. The chosen time period covers the onset of the Omicron wave, aligning with this study’s focus on evaluating methods for estimating $R_{0}(t)$ during abrupt changes in transmission rates. We assume a constant case ascertainment rate of 28.37% and a mean infectious period of D=3.5, informed by previous work [34]. To mitigate reporting bias during weekends, a 7-day moving average was applied to the daily case data. Reporting delays, while acknowledged as another source of bias, were not explicitly incorporated in this analysis. Failing to account for reporting delays could potentially lead to an underestimation of reproduction numbers during periods of increasing daily cases. The magnitude of this bias would probably depend on the length and variability of the reporting delays. Furthermore, not accounting for these delays could affect the precision of the $R_{0}(t)$ estimates, potentially widening the confidence intervals around estimates.

Inference was performed with each of the six methods. Because ground truth data for S(t) are unavailable, we compare estimates of $R_{e}(t)$, computed per equation (2.29), with EpiEstim and EpiFilter. Similarly, there is no ground truth $R_{e}(t)$; thus, the performance for each method was evaluated using the posterior predictive distribution and coverage. Coverage was computed as the proportion of actual case counts that fall within the 95% CrI of the posterior predictive distribution.

**Figure 4.**
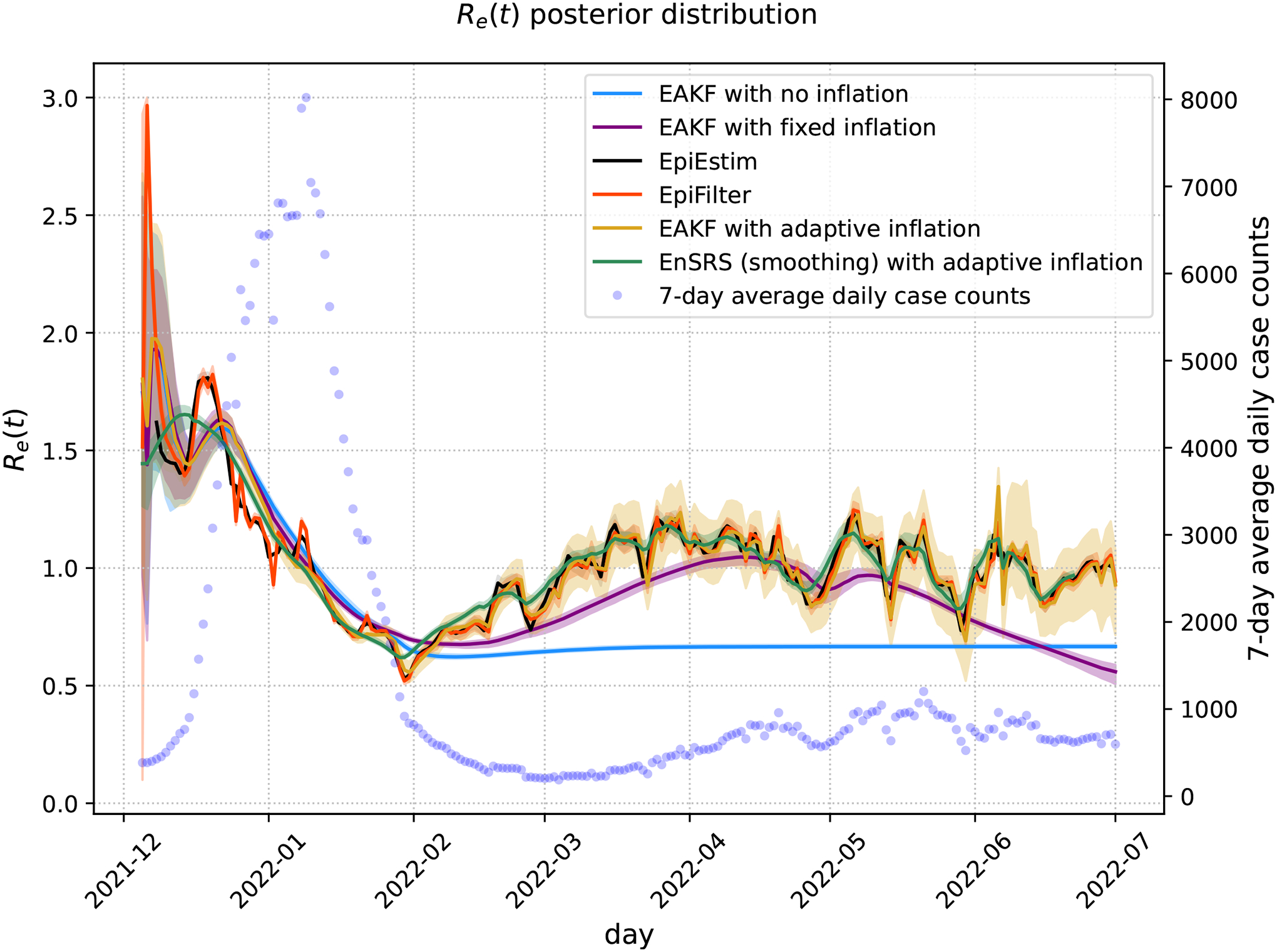
**$R_{e}(t)$** posterior distribution when fit to New York County case data. The posterior mean of each method is presented with 95% Crl, represented by the shaded region. Also shown on the right y-axis is the 7-day moving average of daily cases in Manhattan.

Figure 4 presents the posterior mean and 95% CrI as estimated by each method along with the 7-day moving average of daily cases adjusted for the ascertainment rate. The EAKF with no inflation experiences filter divergence as expected. The EAKF with fixed inflation produces estimates that are consistent with the other EAKF methods until early February 2022, after which its trajectory correlates with the overall trend of the daily cases counts. A strong agreement was observed between the mean estimates of the EAKF and EnSRS with adaption inflation along with EpiEstim and EpiFilter. However, the spread in the estimates was greatest for the EAKF with adaptive inflation followed by the EnSRS, EpiEstim and EpiFilter.

The challenge of accurately quantifying uncertainty is a central theme in $R_{0}(t)$ estimation. Methods like EpiEstim rely on parameters such as the time window to control variance, a choice that often involves a trade-off between smoothness and responsiveness. Similarly, EpiFilter uses a smoothing parameter to influence variance. The adaptive inflation techniques explored in our study offer a distinct, data-driven approach to managing ensemble variance dynamically. The key advantage is that the inflation factor has the flexibility to change over the course of the epidemic in response to how well the model fits new data, rather than relying on a single, fixed parameter. A formal study exploring the relationship between the EAKF inflation factor and the window size or smoothing parameters in these other methods would be a valuable area for future work.

**Figure 5.**
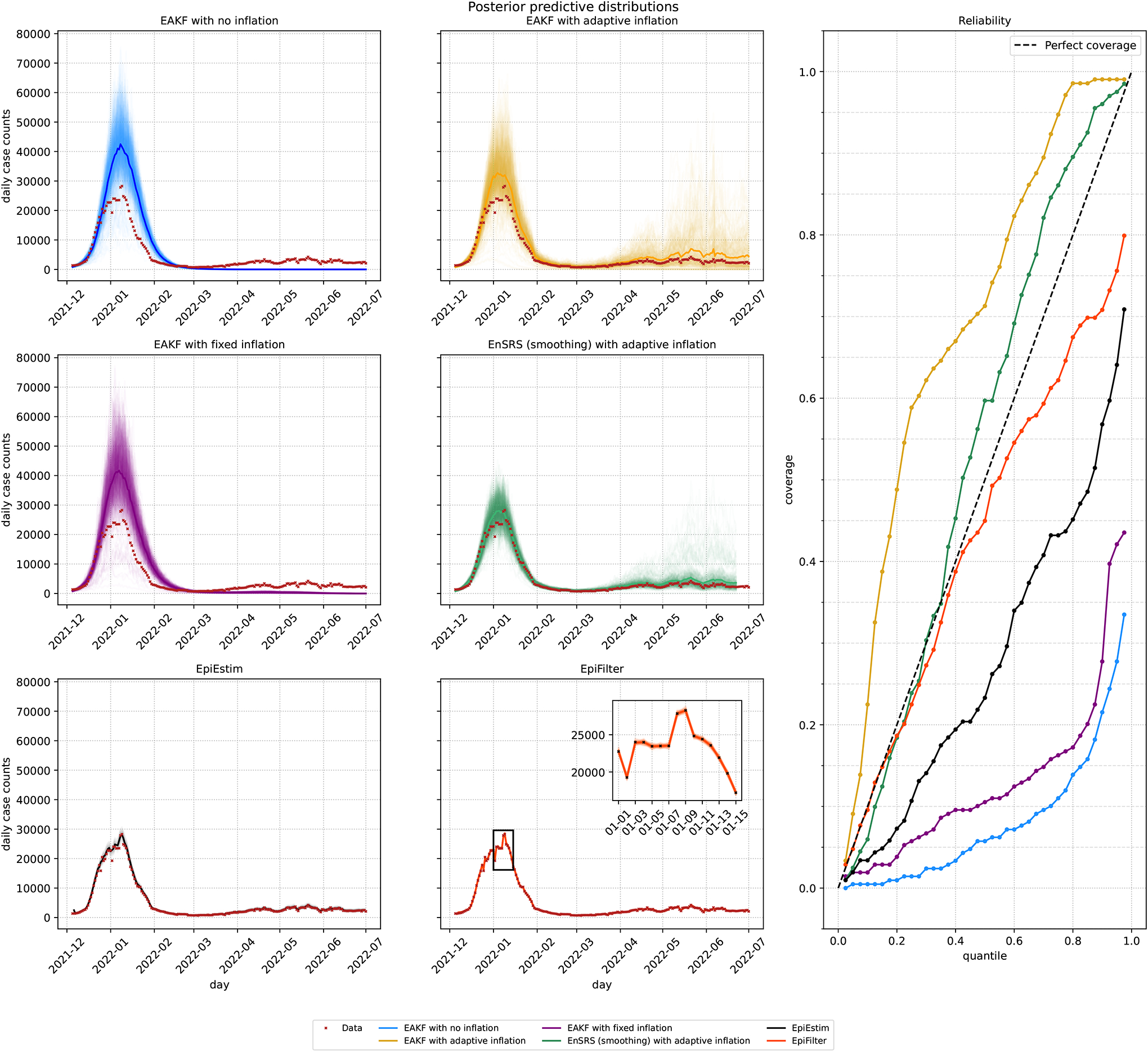
Posterior predictive distribution and reliability for New York County case data. Posterior predictive distributions for each of the six methods. Each line in the posterior predictive distribution represents one realization. Each solid line represents the mean of the posterior predictive distribution. The data in the inset axes for EpiFilter is shown in black for better visibility. On the right are the coverage probabilities for each method.

Figure 5 presents the posterior predictive distributions and reliability plot for each method. The posterior predictive distributions for the EAKF and EnSRS methods were generated using a stochastic SIR model using the posterior estimates of $R_{e}(t)$ with $S_{0}=526\;680$ and $I_{0}=2000$. The posterior predictive distribution for EpiEstim and EpiFilter were performed using the underlying renewal equation model with $i_{0}=2500$ and a window of Δ=3. Inference for EpiFilter was performed using η=0.02. We performed a grid search to identify the initial condition, time window and smoothing hyperparameter that produced the best coverage over the following intervals: $i_{0}\in [1500,3600],\Delta \in [3,10],\eta \in [0.01,0.5]$. The EAKF with no inflation and the EAKF with fixed inflation demonstrate poor coverage. Both overestimate the peak of the first wave and have no activity afterwards due to $R_{e}(t)$ estimates being below 1 for the no inflation method or not increasing fast enough for the fixed inflation method. By contrast, the EAKF, the EnSRS with adaptive inflation and EpiEstim exhibit significantly better coverage. The reliability plot shows that the EAKF with adaptive inflation is slightly underconfident, as the coverage plot is slightly above the identity line. By contrast, EpiEstim is slightly overconfident, with coverage slightly below the identity line. EpiFilter produces overconfident estimates of $R_{e}(t)$ that closely track the data in which the 97.5% quantile captures less than 80% of the data. Steyn & Parag [17] note that the default hyperparameter values of EpiFilter can negatively impact inference of $R_{e}(t)$ by potentially producing overconfident estimates with artificially narrow CrIs. The EnSRS with adaptive inflation provides the best coverage and is close to being perfectly calibrated.

It is important to acknowledge that as a fixed-lag smoother, the EnSRS is not designed for real-time estimation of $R_{0}(t)$. However, we contend that a lag of l=10 days represents a reasonable trade-off between timeliness and accuracy for many public health applications. Reporting delays inherent in surveillance systems often mean that finalized case data are only available after a comparable period, making the more precise, smoothed estimates valuable for retrospective analysis and informed policy evaluation.

While this comparison using real data illustrates the advantages of using the EAKF with adaptive inflation, it precludes definitive conclusions regarding these $R_{e}(t)$ estimations. Potential bias in the data, including time-varying underreporting and reporting delays, warrants explicit incorporation into the model framework. Additionally, addressing model misspecification and population heterogeneity is essential for generating more robust and realistic estimates of $R_{e}(t)$.

## 4. Conclusion

In this study, we evaluated the performance of six methods for estimating the time-varying basic reproduction number, $R_{0}(t)$, using synthetic data generated from a stochastic SIR model and empirical COVID-19 case data. The methods employed included the EAKF with no inflation, fixed inflation and adaptive inflation, the EnSRS with adaptive inflation, EpiEstim and EpiFilter. Our results demonstrate that the EAKF and EnSRS methods with adaptive inflation outperform other approaches in accurately capturing the true $R_{0}(t)$, particularly in scenarios with abrupt changes in transmission rates. This enhanced parameter inference leads to more realistic model simulations that correctly capture the timing and amplitude of the resulting epidemic waves, highlighting the value of these methods for real-time tracking and forecasting. The adaptive inflation techniques effectively address the challenges of covariance underestimation and filter divergence, leading to more robust and reliable estimates of $R_{0}(t)$.

In this study, we focused on a sigmoid-driven change in $R_{0}(t)$. Future work should extend this evaluation to other dynamic scenarios, such as applying step-like changes to reflect control measures or using sinusoidal functions to capture seasonality. Doing so would more rigorously test the practical benefits of these adaptive methods across diverse epidemiological situations. Furthermore, the adaptive inflation algorithm itself could be refined. The method we employed assumes a Gaussian prior for the inflation factor, which does not enforce that the factor must be positive. Future research could explore the use of priors that are strictly positive, such as a lognormal or truncated normal distribution, which may offer a more theoretically robust foundation for covariance inflation.

The findings of this study have important implications for public health officials and epidemiologists. The accurate estimation of $R_{0}(t)$ is crucial for understanding the dynamics of disease transmission, evaluating the effectiveness of interventions and making informed decisions about resource allocation and public health policies. The superior performance of the EAKF and EnSRS methods with adaptive inflation suggests that these methods should be considered as valuable tools for real-time monitoring and prediction of infectious disease outbreaks. By providing more accurate and reliable estimates of $R_{0}(t)$, these methods can contribute to more effective and timely public health responses, ultimately leading to better control and mitigation of infectious diseases.

## Supplementary Material

Supp Info

Electronic supplementary material is available online at https://doi.org/10.6084/m9.figshare.c.8183849.

## Figures and Tables

**Table 1.** EAKF notation.

variable	definition
${\hat{v}}_{j+1}^{\left(m\right)}$	prior ensemble member m at time $t_{j+1}$
${\hat{y}}_{j+1}^{\left(m\right)}$	prior ensemble member m of observation at time $t_{j+1}$
${\hat{m}}_{j+1}$	prior ensemble mean at time $t_{j+1}$
${\hat{C}}_{j+1}$	prior ensemble covariance matrix at time $t_{j+1}$
${\hat{\sigma }}_{j+1}^{2}$	prior ensemble variance of observed variable at time $t_{j+1}$
$\gamma_{j+1}$	observation error variance (OEV)
α	$\sqrt{\gamma_{j+1}{\left(\gamma_{j+1}+{\overset{ˆ}{\sigma }}_{j+1}^{2}\right)}^{-1}}$
$v_{j+1}^{\left(m\right)}$	posterior ensemble member m at time $t_{j+1}$
$y_{j+1}^{\left(m\right)}$	posterior observation ensemble member m at time $t_{j+1}$
$m_{j+1}$	posterior ensemble mean at time $t_{j+1}$
$C_{j+1}$	posterior ensemble covariance matrix at time $t_{j+1}$
H	observation operator
$y_{j+1}^{†}$	observation at time $t_{j+1}$
$v_{j+1}$	model state and parameter random vector at time $t_{j+1}$
$y_{j+1}$	observation random variable at time $t_{j+1}$

**Table 2.** Performance metrics (end of the second epidemic).

method	$R_{0}(t)$ RMSE	$W_{2}(p,q)$	$D_{KL}(p\|q)$	CRPS
EAKF no inflation	1.17 (0.67, 1.88)	0.86 (0.4, 1.29)	6.6 (2.19, 11.48)	186.33 (78.91, 328.76)
EAKF fixed inflation	0.53 (0.24, 1.28)	0.74 (0.29, 1.16)	2.9 (0.37, 7.76)	92.63 (19.09, 296.69)
EAKF adaptive inflation	0.39 (0.24, 0.75)	0.52 (0.26, 0.82)	0.81 (0.27, 2.21)	34.78 (17.37, 69.62)
EnSRS adaptive inflation	0.19 (0.09, 0.51)	0.34 (0.18, 0.69)	0.86 (0.3, 3.63)	61.91 (30.24, 120.13)

Performance metrics for posterior predictive distributions p and data distribution q. All metrics were computed up to the end of the second epidemic when daily new cases reached zero.

**Table 3.** Performance metrics (beginning of the second epidemic).

method	$R_{0}(t)$ RMSE	$W_{2}(p,q)$	$D_{KL}(p\|q)$	CRPS
EAKF no inflation	0.27 (0.19, 0.4)	0.62 (0.29, 0.98)	1.24 (0.47, 2.64)	38.52 (17.44, 79.33)
EAKF fixed inflation	0.31 (0.24, 0.41)	0.55 (0.3, 0.84)	0.83 (0.34, 1.78)	36.16 (16.58, 73.8)
EAKF adaptive inflation	0.36 (0.27, 0.54)	0.52 (0.29, 0.77)	0.66 (0.29, 1.43)	31.72 (15.36, 64.44)
EnSRS adaptive inflation	0.11 (0.06, 0.21)	0.30 (0.2, 0.41)	0.83 (0.39, 1.53)	59.46 (28.81, 116.54)

Performance metrics for posterior predictive distributions p and data distribution q. All metrics were computed up to the beginning of the second epidemic when daily new cases begin to increase again.

## Data Availability

All code for analysis and figure generation is available at [35]. Supplementary material is available online [36].
