# Supplementary material for: Data assimilation for estimating time-varying reproduction numbers
Source: J R Soc Interface. Author manuscript; Available in PMC 2026 Apr 13. (PMC13075726; doi:10.1098/rsif.2025.0131)
Supplement: Supp Info [file NIHMS2161645-supplement-Supp_Info.pdf]

Text S1

Han Yong Wunrow[1*], Sen Pei[2], Jeffrey Shaman[2,3], Marc W. Spiegelman[1,4]

[1]Department of Applied Physics and Applied Mathematics, Columbia University, New York, New York, United States of America
[2]Department of Environmental Health Sciences, Mailman School of Public Health, Columbia University, New York, New York, United States of America
[3]Columbia Climate School, Columbia University, New York, New York, United States of America
[4]Department of Earth and Environmental Sciences, Columbia University, New York, New York, United States of America
*nhw2114@columbia.edu

Multivariate EAKF

Here we introduce the Ensemble Adjustment Kalman Filter (EAKF) in the more general multivariate case. This would be applicable in settings where observations are multivariate (e.g., case counts across multiple locations). Consider the stochastic dynamics model given by

$$\begin{aligned} \boldsymbol{v}_{j+1} &= \Psi\left(\boldsymbol{v}_j\right), j = 0, \ldots, T-1 \\ \boldsymbol{v}_0 &\sim \mathcal{U}\left(a, b\right), \end{aligned} \tag{1}$$

where $\boldsymbol{v}_j$ represents our model states and parameters at time t_j. Note that our model omits the Gaussian noise term and employs a uniform prior for $\boldsymbol{v}_0$, deviating from the traditional EAKF's Gaussian prior. Now consider the data model given by

$$\begin{aligned} \boldsymbol{y}_{j+1} &= \boldsymbol{H}\boldsymbol{v}_{j+1} + \eta_{j+1},\ j = 0, \ldots, T-1, \\ \boldsymbol{\eta}_{j+1} &\sim \mathcal{N}(0, \boldsymbol{\Gamma}_{j+1}), \end{aligned} \tag{2}$$

where $\boldsymbol{y}_{j+1}$ is our observation at time t_{j+1}, $\boldsymbol{H}$ is our linear observation operator that maps the latent model state into observation space, and $\boldsymbol{\eta}_{j+1}$ represents observation noise. In equations 1 and 2, both $\boldsymbol{v}_j$ and $\boldsymbol{y}_j$ are random vectors. The EAKF approximates the posterior distributions $\mathbb{P}(\boldsymbol{v}_j \mid \{\boldsymbol{y}_1, \ldots, \boldsymbol{y}_j\})$ using an ensemble of M members, denoted by $\boldsymbol{v}_j^{(m)}$ where $m = 1, \ldots, M$. Each ensemble member is equally weighted and represents a possible state of the system at time t_j.

The EAKF algorithm can be decomposed into two steps:

$$\text{Prior/ Forecast}\begin{cases}\widehat{\boldsymbol{v}}_{j+1}^{(m)} &= \Psi\left(\boldsymbol{v}_j^{(m)}\right),\ m=1,\ldots,M\\ \widehat{\boldsymbol{m}}_{j+1} &= \frac{1}{N}\sum_{n=1}^{N}\widehat{\boldsymbol{v}}_{j+1}^{(m)},\\ \widehat{\boldsymbol{C}}_{j+1} &= \frac{1}{N-1}\sum_{n=1}^{N}\left(\widehat{\boldsymbol{v}}_{j+1}^{(m)}-\widehat{\boldsymbol{m}}_{j+1}\right)\left(\widehat{\boldsymbol{v}}_{j+1}^{(m)}-\widehat{\boldsymbol{m}}_{j+1}\right)^\top\end{cases} \tag{3}$$

$$\text{Posterior/ Analysis}\begin{cases}\boldsymbol{v}_{j+1}^{(m)} &= \boldsymbol{A}\left(\widehat{\boldsymbol{v}}_{j+1}^{(m)}-\widehat{\boldsymbol{m}}_{j+1}\right)+\boldsymbol{m}_{j+1}\\ \boldsymbol{m}_{j+1} &= \boldsymbol{C}_{j+1}\left(\widehat{\boldsymbol{C}}_{j+1}^{-1}\widehat{\boldsymbol{m}}_{j+1}+\boldsymbol{H}^\top\boldsymbol{\Gamma}_{j+1}^{-1}\boldsymbol{y}_{j+1}^\dagger\right)\\ \boldsymbol{C}_{j+1} &= \boldsymbol{A}\widehat{\boldsymbol{C}}_{j+1}\boldsymbol{A}^\top\\ \boldsymbol{A} &= \widehat{\boldsymbol{C}}_{j+1}^{1/2}\boldsymbol{Q}(\boldsymbol{I}+\boldsymbol{\Lambda})^{-1/2}\boldsymbol{\Sigma}^{+}\boldsymbol{U}^\top\end{cases} \tag{4}$$

where $\boldsymbol{U}\boldsymbol{\Sigma}\boldsymbol{V}^\top = \widehat{\boldsymbol{C}}_{j+1}^{1/2}$ is the full singular value decomposition, $\boldsymbol{Q}\boldsymbol{\Lambda}\boldsymbol{Q}^{-1} = \left(\boldsymbol{H}\widehat{\boldsymbol{C}}_{j+1}^{1/2}\right)^\top \boldsymbol{\Gamma}_{j+1}^{-1}\left(\boldsymbol{H}\widehat{\boldsymbol{C}}_{j+1}^{1/2}\right)$ is an eigendecomposition, and $\boldsymbol{\Sigma}^{+}$ is the pseudoinverse of $\boldsymbol{\Sigma}$.

**Variable**	**Definition**
$\widehat{\boldsymbol{v}}_{j+1}^{(m)}$	Prior ensemble member m at time t_{j+1}
$\widehat{\boldsymbol{m}}_{j+1}$	Prior ensemble mean at time t_{j+1}
$\widehat{\boldsymbol{C}}_{j+1}$	Prior ensemble covariance matrix at time t_{j+1}
$\boldsymbol{v}_{j+1}^{(m)}$	Posterior ensemble member m at time t_{j+1}
$\boldsymbol{m}_{j+1}$	Posterior ensemble mean at time t_{j+1}
$\boldsymbol{C}_{j+1}$	Posterior ensemble covariance matrix at time t_{j+1}
$\boldsymbol{H}$	Observation operator
$\boldsymbol{y}_{j+1}^\dagger$	Observation at time t_{j+1}
$\boldsymbol{\Gamma}_{j+1}$	Observation error covariance matrix at time t_{j+1}
$\boldsymbol{v}_{j+1}$	Model and parameter random vector at time t_{j+1}
$\boldsymbol{y}_{j+1}$	Observation random vector at time t_{j+1}

Table S1: Multivariate EAKF Notation.

A key property of the EAKF is that the adjustment matrix $\boldsymbol{A}$ in equation 4 is chosen to satisfy the covariance update equation of the Kalman Filter:

$$\boldsymbol{C}_{j+1} = \left[\widehat{\boldsymbol{C}}_{j+1}^{-1}+\boldsymbol{H}^\top\boldsymbol{\Gamma}_{j+1}^{-1}\boldsymbol{H}\right]^{-1}. \tag{5}$$

Cumulative Average of Performance Metrics

To further compare the four methods, we computed the cumulative average for each performance metric across time, averaged over all parameter scenarios shown in figure S1. Taking the cumulative average of each performance metric is a way to compare the methods' performance up to a particular time. It is important to note that the time scales vary across parameter scenarios, so the change in relative difference in cumulative average between methods may occur at different times for each scenario. We see that the EAKF and EnSRS with adaptive inflation have the best performance with the lowest cumulative average on the last days. The EAKF with no inflation consistently performs the worst with the highest cumulative average across all metrics.

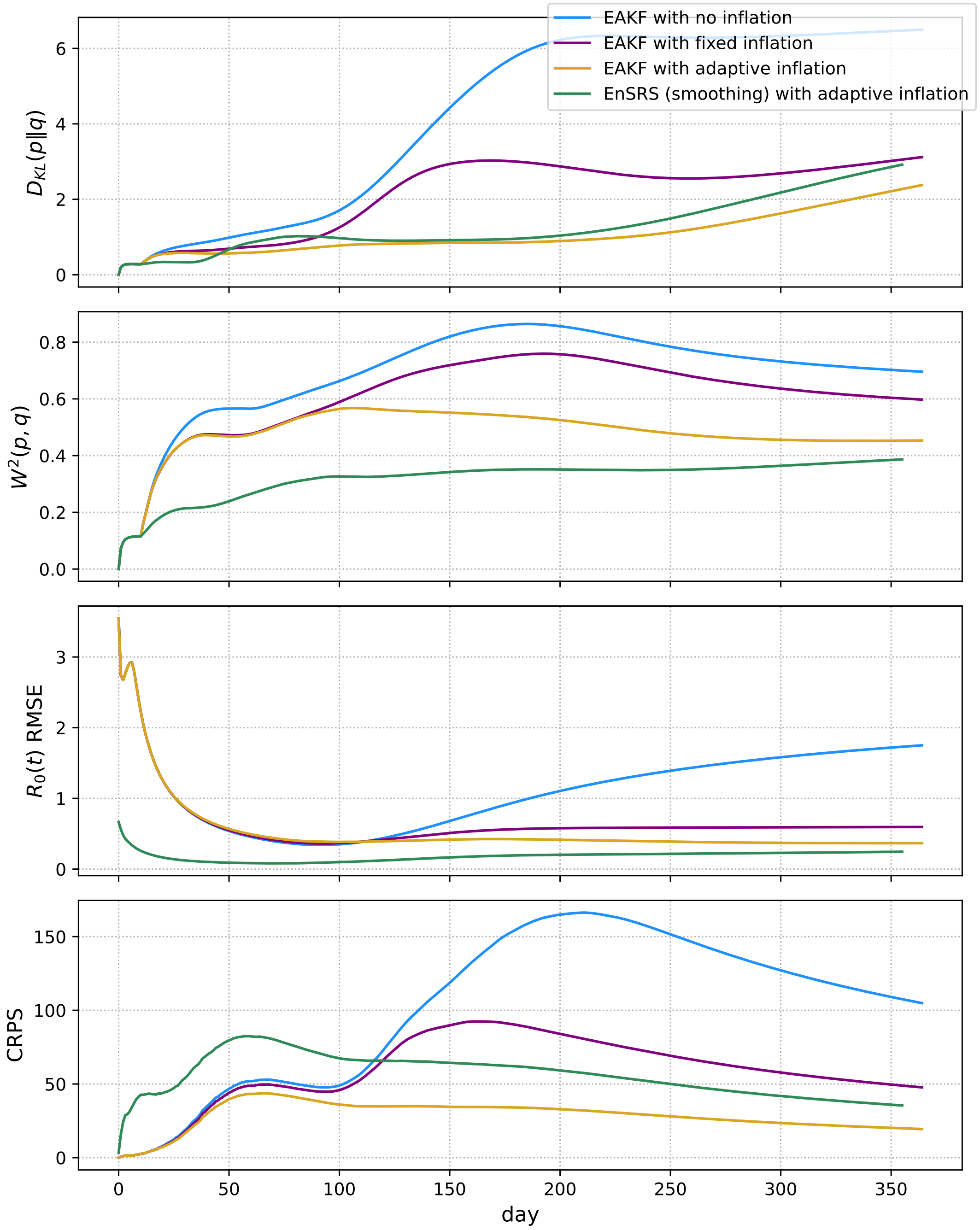

Figure S1: **Performance metrics cumulative average.** Cumulative average across time for each performance metric averaged over all parameter scenarios.

SIR versus Renewal Equation Synthetic Data

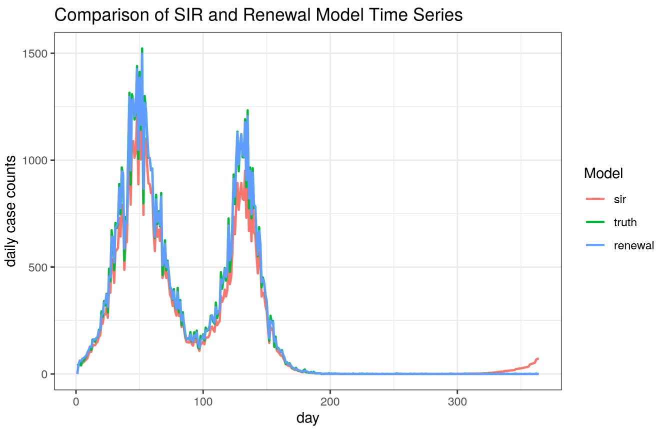

Figure S2: Comparison of data generated using the renewal equation vs SIR model with the same inferred $R_e(t)$. The sigmoid parameters for this example were $\beta_0 = 0.35$, $\beta_1 = 0.95$, $t_{mid} = 108$, $k = 0.2$.

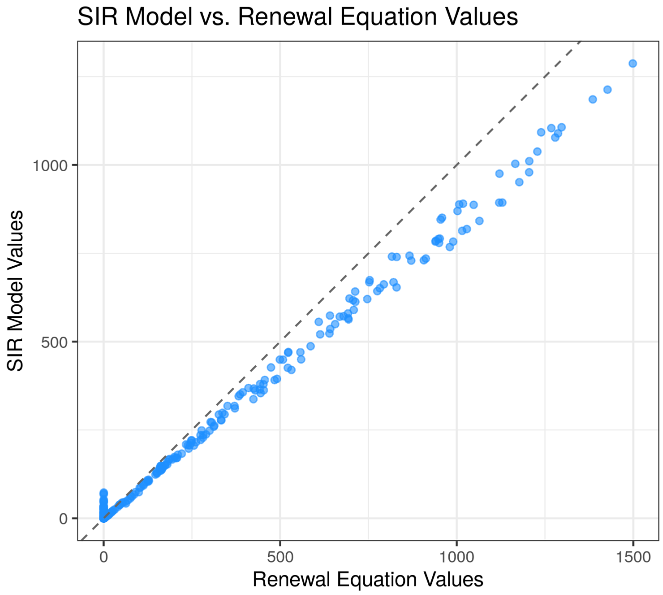

Figure S3: Comparison of data generated using the renewal equation vs SIR model with the same inferred $R_e(t)$. The sigmoid parameters for this example were $\beta_0 = 0.35$, $\beta_1 = 0.95$, $t_{mid} = 108$, $k = 0.2$.

EpiFilter Grid Search

Figure S4 evaluates the calibration of the EpiFilter method's estimates with varying values of η using empirical data. The dashed diagonal line represents perfect calibration, where the empirical coverage matches the nominal coverage. The plot clearly demonstrates that the model's calibration is sensitive to the choice of η. For $\eta = 0.02$, the model is well-calibrated with empirical coverages closely matching the nominal coverage. As η increases, the method becomes progressively miscalibrated, with the empirical coverage falling above the nominal level. For instance, with $\eta = 0.1$, a nominal coverage 25% achieves an empirical coverage of more than 85%.

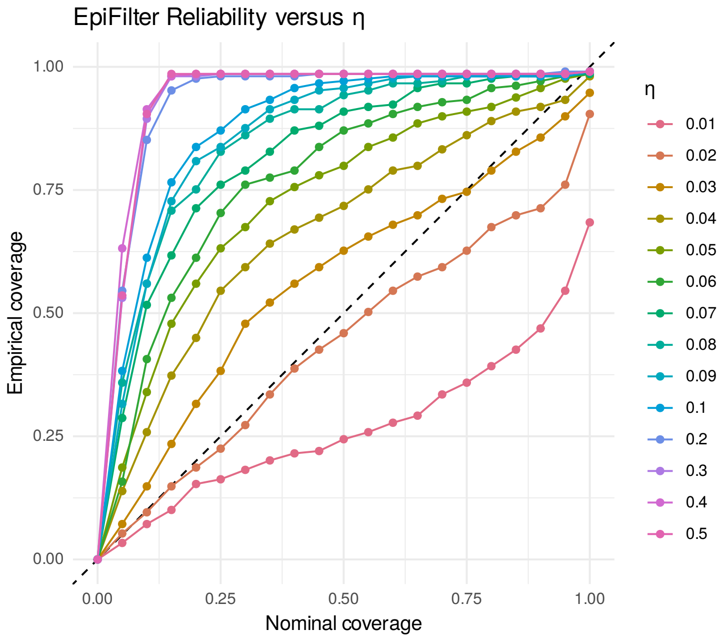

Figure S4: Reliability plot for EpiFilter on empirical data with varying value of η
